# Nitric Oxide in Fungi: Production and Function

**DOI:** 10.3390/jof10020155

**Published:** 2024-02-15

**Authors:** Nan-Nan Yu, Gyungsoon Park

**Affiliations:** 1 Plasma Bioscience Research Center, Department of Plasma-Bio Display, Kwangwoon University, Seoul 01897, Republic of Korea; nannan19950326@163.com; 2 Department of Electrical and Biological Physics, Kwangwoon University, Seoul 01897, Republic of Korea

**Keywords:** nitric oxide, fungi, endogenous production, nitric oxide synthase, nitrite reductase, nitrate reductase, biological function, signaling molecule

## Abstract

Nitric oxide (NO) is synthesized in all kingdoms of life, where it plays a role in the regulation of various physiological and developmental processes. In terms of endogenous NO biology, fungi have been less well researched than mammals, plants, and bacteria. In this review, we summarize and discuss the studies to date on intracellular NO biosynthesis and function in fungi. Two mechanisms for NO biosynthesis, NO synthase (NOS)-mediated arginine oxidation and nitrate- and nitrite-reductase-mediated nitrite reduction, are the most frequently reported. Furthermore, we summarize the multifaceted functions of NO in fungi as well as its role as a signaling molecule in fungal growth regulation, development, abiotic stress, virulence regulation, and metabolism. Finally, we present potential directions for future research on fungal NO biology.

## 1. Introduction

Nitric oxide (NO) is a diatomic gas synthesized by bacteria, fungi, plants, and mammals. Although the mechanisms for NO biosynthesis vary among species, there is increasing evidence demonstrating the conserved role of endogenous NO as a signaling molecule that regulates numerous physiological and differential processes [1,2,3]. In mammals, NO is produced by NO synthase (NOS), which plays a crucial role in vasodilation, neurotransmission, and the immune response [4,5]. NO produced by endothelial cells located within blood vessels induces vasodilation, increases blood flow, and regulates blood pressure [6]. In the nervous system, neuron-produced NO acts as a neurotransmitter, facilitates in synaptic transmission and plasticity, and ultimately affects learning and memory processes [7]. During immune responses, immune-cell-produced NO enhances the antimicrobial activity of macrophages and regulates the expression of inflammatory factors and chemokines [8,9].

In plants, NO is an important signaling molecule that regulates plant growth, maturation, and stress as well as seed germination, root formation, stomatal aperture, flowering, and senescence [3,10,11,12]. During embryonic development, NO participates in seed dormancy and germination by regulating protein tyrosine nitration and cysteine *S-*nitrosylation [13]. The root cells at the root tip also generate NO, which is implicated in root hair development and lateral root formation [14,15]. The stomatal opening and closing, gas exchange, and water loss can be controlled by regulating the NO levels in guard cells [16]. NO interacts with plant hormones (auxins, abscisic acid, and gibberellins) to regulate plant growth and development [13,15,16]. However, NO synthesis does not appear to follow the same pathway in plant cells as in mammalian cells. Although studies have demonstrated the existence of NOS-like enzyme activity in plants, there is low sequence homology between plant and mammalian NOS [17,18]. The amino acid sequence of NOS in photosynthetic green algae (*Ostreococcus tauri*) is 45% similar to that of human NOS [19]. The level of NO increases after high-intensity light irradiation and the addition of L-arginine, indicating the existence of arginine-dependent NO production in plant cells [19]. In addition, an NR (nitrate reductase)–NOFNiR (nitric-oxide-forming nitrite reductase)-dependent NO synthesis pathway has been discovered in plants. In this process (NO_3_^−^ → NO_2_^−^ → NO), NR mediates the transfer of electrons generated during the reduction of NO_3_^−^ to NO_2_^−^ to the partner protein, mARC (mitochondrial amidoxime reducing component)/NOFNiR [20]. mARC/NOFNiR utilizes the electrons received from NR to reduce NO_2_^−^ to NO, and this electron transfer is a key step in this NO synthesis pathway [20,21].

NO production has also been observed in prokaryotic bacterial cells [22,23]. Bacterial NO is generated via nitrite (NO_2_^−^) reduction by nitrite reductase during denitrification and via ammonia (NH_3_) oxidation by hydroxylamine (NH_2_OH) oxidoreductase [24,25]. In addition, regions homologous to mammalian NOS oxygenase domains have been found in the genomes of many bacteria, and bacterial NOS can mediate arginine oxidation to produce NO [26,27,28,29]. In bacterial cells, endogenous NO regulates pathogenicity, toxin biosynthesis, and morphological differentiation [28,30].

Compared with other organisms, fungi have received less attention with respect to endogenous NO production and function [2]. In recent years, there has been an increase in experimental data demonstrating that fungi can produce endogenous NO, which may be involved in fungal physiology, cell differentiation, and pathogenicity regulation [2,31,32]. NO appears to be a universal signaling molecule conserved in organisms of all kingdoms. However, the biosynthetic pathways and functions of endogenous NO in fungal cells are not fully understood [2,31]. Fungi exhibit species diversity and functional complexity, which may lead to various aspects of NO production and function [33,34]. In this review, we summarize the findings of studies on the functions and mechanisms of NO production in various fungi. Endogenous NO is likely to be a universal signaling molecule that is well conserved in all organisms. To understand the universal and conserved roles and fate of NO in prokaryotic and eukaryotic cells, it is important to review the current literature.

## 2. Fungal Endogenous NO Generation and Removal

The details of NO biosynthesis within fungal cells have not yet been clearly elucidated. In fungal genomes, gene sequences that are highly homologous to mammalian NOS are rarely found. However, NOS-like activity has been observed in fungal cells through measuring enzyme activity or using mammalian NOS enzyme inhibitors [2,32]. Like plants, fungi are likely to have NOS-independent mechanisms for NO biosynthesis, such as nitrite reduction by nitrite reductase during denitrification. However, the different molecular structures of the putative NOS proteins and other NOS-independent mechanisms indicate that further studies should be performed to better understand NO biosynthesis in fungi.

### 2.1. Arginine-Dependent NO Formation

L-Arginine can be oxidized to L-citrulline and NO via NOS [35]. NOS-mediated NO synthesis is well characterized in mammalian cells [5]. Enzymes homologous to mammalian NOS have been found in plant, bacterial, and fungal genomes; however, they possess low sequence homology to mammalian NOS [18,22,32]. The involvement of NOS in NO synthesis in fungi has been examined by measuring biochemical enzyme activity and inhibiting enzyme activity (Table 1), where enzyme activity was assessed by determining the L-arginine to L-citrulline rate of conversion [36,37,38,39,40,41,42,43,44,45,46,47]. NO synthase activity can reach 500 pmol/mg/min in the fruiting bodies of *Flammulina velutipes* [37], whereas it is only 3 and 18 pmol/mg/min in the mycelia of *Phycomyces blakesleeanus* and *Neurospora crassa*, respectively [48]. Mammalian NOS inhibitors such as L-NAME (N^G^-nitro-L-arginine methyl ester), L-NMMA (N^G^-methyl-L-arginine acetate salt), L-NNA (N_w_-nitro-L-arginine), and AG (aminoguanidine) reduce intracellular NO levels, indicating the involvement of NOS in NO synthesis [36,38,42,45,48,49,50,51,52,53,54]. In several fungi, NOS-dependent NO production only occurs under specific environmental conditions. For example, intracellular NO levels in *Pleurotus eryngii* var. *tuoliensis* increase along with NOS activity under heat stress [53].

A fungal genome analysis has revealed that NOS-like genes with high sequence homology to mammalian NOS are rarely found in the fungal genome. However, in some recent studies, NOS proteins were purified from *F. velutipes* and *S. cerevisiae* using affinity chromatography [36,37], and NOS genes were identified in the genomes of *Shiraia* sp. Slf14, *M. phaseolina*, and *I. obliquus* [42,54,62]. Fungal NOSs have a degree of homology or functional similarity to mammalian NOS but may differ significantly in structure, regulation, and substrate specificity [32,63]. In fungi, NOS-like enzymes are highly regulated and influenced by various environmental factors, including changes in oxygen and cofactor levels [32]. In *Aspergillus nidulans*, the addition of L-arginine to liquid culture media induces a burst of intracellular NO, a process that is inseparable from the action of NOS [45]. NO production was controlled by the level of the available L-arginine in the cell, which was regulated by mobilization from the vacuole, not by the urea cycle [45].

### 2.2. Nitrite (NO_2_^−^)-Dependent NO Formation 

In eukaryotes such as plants, microalgae, and mammals, NO can be synthesized via nitrite (NO_2_^−^) reduction through catalysis of the mitochondrial amidoxime-reducing component (mARC), also referred to as nitrite reductase [20,21,64,65]. All mARC enzymes need a partner protein with reducing power, for which plant mARC uses nitrate reductase (NR) [20,21]. In plants and microalgae, NO is produced during nitrate (NO_3_^−^) assimilation; NO_3_^−^ taken up into the cell is reduced to NO_2_^−^ by the action of nitrate reductase (NR), a partner protein of mARC, and further reduced to NO through the catalysis of mARC, also referred to as nitric-oxide-forming nitrite reductase (NOFNiR), finally converting to ammonium [20,21]. In prokaryotic bacteria, NO is generated during denitrification (NO_3_^−^ → NO_2_^−^ → NO → N_2_O → N_2_), an anaerobic respiration process in which electrons from the mitochondrial respiratory chain are transferred to nitrogen oxides (the final electron acceptors), leading to reduction of NO_3_^−^ and NO_2_^−^ ultimately to N_2_ [66]. During this process, NO is generated from NO_2_^−^ reduction through catalysis of nitrite reductase (NiR), which is homologous to mARC [66]. 

Similar to bacteria, fungi produce NO during the denitrification process [67,68,69]. Electrons from the respiratory electron transport chain are transferred from donor molecules to NO_3_^−^ through NR catalysis, leading to the reduction to NO_2_^−^ (2NO_3_^−^ + 2H^+^ + 2e^−^ → 2NO_2_^−^ + H_2_O) and then to NO_2_^−^ through NiR catalysis, leading to the reduction to NO (2NO_2_^−^ + 2H^+^ + 2e^−^ → 2NO + H_2_O) [58,69,70,71,72,73,74]. The involvement of NR in fungal NO production has been experimentally demonstrated in several fungi (Table 1). *F. graminearum* senses host signals and triggers NR-dependent NO production during the infection of plant roots [57]. Meanwhile, the produced NO can also directly or indirectly regulate the expression of genes related to fungal virulence and development by regulating the transcriptome [57]. In *A. nidulans*, the NR gene, niaD, is essential for NO production from the vegetative to early developmental stages [55]. *G. lucidum* can also produce NO via NR with methyl jasmonate induction [59]. In the endophytic fungus *Shiraia* sp. Slf14, NR activity and expression are enhanced by an increase in L-arginine levels, promoting NO production [46]. Nitrite reductase (NiR) genes, homologs of mARC, have been identified in many fungi, including *C. tonkinense* (*NirK*) [68], *F. oxysporum* (*NirK* and *Cu-NiR*) [58,68], *Pisolithus* sp.1 (*NiR*) [75], *M. phaseolina* (*EKG10021.1*) [64], and *A. niger* (*CAK45930.1* and *SPB51236.1*) [64]. Fungal NiR is associated with the mitochondrial respiratory electron chain and structurally similar to copper-containing NirK (NiR) in bacteria [58]. The involvement of fungal NiR in NO production has been demonstrated based on the upregulation of NiR transcripts in the *Preussia* sp. BSL-10 [60] and purification of the enzyme and measurement of its activity in *C. tonkinense* and *F. oxysporum* [56,58,68]. In *S. cerevisiae*, NO_2_^−^-dependent NO production occurs only under hypoxic conditions [61]. This may be because NR and NiR expression and activity are upregulated during denitrification under hypoxic conditions, as observed in *F. oxysporum* [76].

Although NO_2_^−^-dependent NO generation is closely associated with respiratory processes, non-respiratory NO formation has also been observed in fungi. When *F. graminearum* infects a plant root, NO is generated within fungal cells during host recognition prior to contact with the plant root, and host signals seem to trigger the expression of NR [57]. 

### 2.3. Other NO Formation Pathways and Regulation of NO Homeostasis in Fungi

Studies show that fungal NO can be produced through non-enzymatic processes. In the rice blast fungus *Magnaporthe oryzae*, the deletion of NOS, NR, and NiR genes does not affect NO production [77]. This indicates that other enzymatic or non-enzymatic NO generation may be possibly present in fungi. Non-enzymatic nitrite (NO_2_^−^) conversion to NO can be promoted under an acidic environment (2 HNO_2_ ↔ NO + NO_2_ + H_2_O ↔ 2NO + 2O_2_ + H_2_O) [78]. There is no evidence of non-enzymatic NO production by fungi to date. However, non-enzymatic NO production has often been observed in the human stomach, oral cavity, skin surface, urine, and plant cytoplasmic apoplasm [78,79,80]. This may be because the pKa of nitrite is approximately 3.2, and the pH values in these areas are <4.5, which is suitable for non-enzymatic NO formation [78,79]. 

NO, a radical, generates dual effects on a cell depending on its intracellular levels. It can act as a signaling molecule at low concentrations and display cytotoxic effects at high concentrations [2,81]. NO can be used as a defense tool for killing pathogens in animal and plant cells and can also act beneficially as a signaling molecule in regulating various cellular processes such as development, vasoconstriction, reproduction, and stress regulation [82,83,84,85]. NO homeostasis is therefore important for maintaining the optimal vitality in organisms, including fungi. 

NO homeostasis can be accomplished by biosynthesis, and metabolism or removal of NO. Fungi have developed effective mechanisms for NO detoxification and removal toward reducing the cytotoxicity caused by an excessive accumulation of endogenous NO. Regarding the fate of NO produced in fungal cells, there are three alternatives. First, NO can be further reduced to N_2_O via catalysis of nitric oxide reductase (Nor), a type of cytochrome P_450_, during the denitrification process [2,69,86]. This mechanism has been demonstrated in the genera *Fusarium*, *Trichoderma*, and *Guehomyces* [31]. Secondly, NO can be converted to less toxic NO_3_^−^ via catalysis of flavohemoglobin NO deoxygenases (FLVs/FHBs) [2,87,88]. The ability of FHB to scavenge NO has been demonstrated in a variety of fungi [2,89,90,91]. Finally, NO can be scavenged by reacting with a cysteine-rich peptide together with S-nitrosoglutathione (GSNO) to generate an S-nitrosated peptide, and the S-nitrosated peptide is denitrosated by S-nitrosoglutathione (GSNO) reductase to be less toxic, as demonstrated in *A. nidulans* [2,69,92]. 

## 3. Function of Endogenous NO and NO Signaling in Fungi

### 3.1. Growth and Development Regulation

Cellularly produced NO is implicated in the regulation of various aspects of fungal growth and development, such as hyphal extension, sporulation, and differentiation (Table 2) [31,32]. Furthermore, it can act as a signaling molecule in developmental processes [81,93]. In *Pleurotus ostreatus* (edible mushrooms), NO negatively regulates the rate of primordium formation by inhibiting the expression and enzymatic activity of mitochondrial aconitase, thereby reducing ATP production [94]. In *A. nidulans*, NO is produced via NR, which is upregulated upon the induction of light-regulated conidiation, and also catabolized by flavohemoglobins [55]. A balance between biosynthesis and catabolism of NO results in NO homeostasis in fungal cells, and deviation from NO homeostasis can serve as a cue for developmental processes [55]. Increases in NO levels reduce conidiation and increase sexual development [55]. The balance between light-induced conidiation (asexual reproduction) and sexual reproduction is influenced by intracellular NO levels via regulating the expression of asexual and sexual developmental regulators [55,88]. A light-dependent change in NOS activity (NO level) is not observed during the regulation of photocarotenogenesis and photoconidiation, and use of NOS inhibitors enhances conidiation in *N. crassa* [44,48]. However, it was also reported in *N. crassa* that high levels of intracellular NO are detected in conidiophores, and the transcription level of genes that are highly expressed during conidiation is reduced upon intracellular NO scavenging [95]. Endogenous NO in *N. crassa* seems to promote hyphal growth, which may be related to the elevated expression of *mss-4* and *gel-3*, as demonstrated in recent studies [95,96]. Other evidence of NO regulation during light-induced development has been demonstrated in *P. blakesleeanus* [38]. In this fungus, light induces macrosporangiophore formation and citrulline production from arginine, processes that are suppressed by NOS inhibitors. In *C. coccodes*, NO was detected in germinating conidia and might regulate conidial germination [49].

Studies have also demonstrated an association between cyclic guanosine monophosphate (cGMP), a downstream molecule generated by NO in mammalian cells, and endogenous NO in fungi. In the aquatic fungus *B. emersonii*, the intracellular NO levels increase during sporulation and are reduced by the addition of an NOS inhibitor. Furthermore, cGMP inhibition prevents zoospore generation [40]. In addition, calcium ions are required for NOS activity [40]. This suggests that the Ca_2_^+^–NO–cGMP signaling pathway, in which NO is synthesized by the mediation of NOS and calcium ions, induces cGMP production, eventually impacting the regulation of zoospore biogenesis. A close association between NOS activity and cGMP levels has also been demonstrated in *C. minitans*, a sclerotial parasite of the plant pathogenic fungus *Sclerotinia sclerotiorum*. In *C. minitans*, L-arginine drives the formation of endogenous NO through NOS, and NO mediates conidia formation [41,50]. In NO-mediated conidiation, cGMP functions as a secondary messenger through the NO–sGC (guanylate cyclase)–cGMP signaling pathway [41]. The pathogenic fungus *C.albicans* can promote its own growth by secreting extracellular vesicles (EVs), finally enhancing pathogenesis [97]. L-Arginine is found to be a key factor in the EV promotion of *C.albicans* growth, and EVs increase the NO level [97]. During the 5-day starvation period needed to induce sporulation competence, NOS expression is strongly upregulated in macroplasmodia of *Physarum polycephalum*, and sporulation competence was inhibited by NOS inhibitors(l-N6–(1-iminoethyl)-lysine (NIL)), indicating the involvement of endogenous NO in sporulation competence [98]. Furthermore, endogenous NO can also regulate fungal growth and development by regulating reactive oxygen species (ROS) levels. During development of a pre-infection state in *Puccinia striiformis Westend* f.sp. *tritici* (*Pst*) (the wheat stripe rust pathogen), NO and ROS serve as key signaling molecules to regulate the polar growth of germ tubes [99]. In *C. albicans*, EVs reduce the intracellular ROS and cell apoptosis by upregulating the expression of the NO dioxygenase gene YHB1 [97].

### 3.2. Response to Stressors

NO acts as a signaling molecule in the fungal response to stress by regulating stress-related gene expression and contributing to cellular defense mechanisms against stress-induced damage (Table 2). Under heat stress, endogenous NO can resist oxidative damage by regulating trehalose accumulation, as has been observed in *P. eryngii* var. *tuoliensis* [53,103]. In *G. lucidum*, the polyamine putrescine alleviates heat shock stress by modulating intracellular NO accumulation, which influences cellular glutamine levels [101]. In addition, researchers found that the expression of a newly discovered gene encoding an inducible NOS-like protein (iNOSL) in *Shiraia* sp. Slf14(w) was significantly increased by heat stress treatment, thereby producing more endogenous NO, and NO can promote the biosynthesis and release of perylenequinones (PQs) [62]. Similarly, under heat shock, high hydrostatic pressure, and hypoxia, there was significantly increased levels of endogenous NO, a response signaling molecule, resulting in the protection of *S. cerevisiae* cells during stress [104,105]. The pH value also has an impact on NO concentration. At pH 3.0, there is a decrease in NO content in the culture media of *L. edodes* and *G. frondosa* [102]. At pH 10.0 (alkaline medium), the NO content increases significantly [102], although it did not change under temperature stress, carbon stress, and nitrogen stress [102]. This seems to imply that NO changes differently under the influence of varied stress factors. In *S. cerevisiae*, NO_2_^−^ dependent NO synthesis is induced by the catalysis of cytochrome c oxidase in mitochondria, regulating the expression of hypoxia-related genes when cells are exposed to hypoxic conditions [61]. H_2_O_2_ (oxidative stress)-induced apoptotic *S. cerevisiae* cells synthesize NO through nitric oxide synthase (NOS)-like activity, and NO mediates GAPDH S-nitrosation, leading to cell death during the chronological lifespan [39]. After stimulation with 420 nm intense pulsed light (IPL), the levels of nitric oxide synthase (NOS) and NO increase, while there are decreases in the intracellular levels of asymmetric dimethylarginine (ADMA), a natural compound structurally similar to L-arginine that acts as an inhibitor of NOS, along with keratinase activity, and fungal growth in *T. rubrum* [43]. Upon exposure to antifungal agents, *A. fumigatus* responds by increasing NO production in the exposed hyphae [100]. Interestingly, the arbuscular mycorrhizal fungus *R. irregularis* can enhance rice NR and NOS activity, increase intracellular NO accumulation in symbionts, and improve the tolerance of rice plants to low-temperature stress by regulating proline metabolism [47]. In conclusion, the different responses triggered by NO in fungi may be related to the different nature of the stress.

In contrast, exogenous NO addition increases the stress tolerance capacity of the fungus. The addition of an NO-producing chemical (sodium nitroprusside, SNP) can improve the resistance of *P. eryngii* var. *tuoliensis* and *Ganoderma oregonense* under high-temperature stress [53,113]. Under metal stress (Cu_2_^+^ or Cd_2_^+^), the addition of exogenous NO exerts a protective effect on *S. cerevisiae* and *P. eryngii* [114,115].

### 3.3. Metabolism Regulation

NO regulates multiple metabolic pathways in fungi, including energy, nitrogen, and secondary metabolite production (Table 2). Many fungal secondary metabolites have been used in medicine, agriculture, and industry, including penicillin (antibiotics from *Penicillium*), cephalosporins (antibiotics from *Acremonium* and *Cephalosporium*), taxanes (anticancer compounds from endophytic fungi), and industrially useful enzymes, such as cellulase, amylase, and flavor/aroma compounds [116]. In the endophytic fungus *Shiraia* sp. Slf14(w), endogenous NO derived from arginine serves as a signaling molecule and can regulate the biosynthesis of secondary metabolite perylenequinones (antimicrobial, anticancer, and antiviral photodynamic therapy agents) via the NO–cGMP–protein kinase G (PKG) signaling pathway [46,62]. In *G. lucidum*, NR-dependent endogenous NO production increases methyl-jasmonate-induced biosynthesis of ganoderic acid, an important secondary metabolite [59]. In extractive *Shiraia* fermentation, elevated levels of endogenous NO significantly increase and regulate the expression of hypocrellin A, a new photosensitizer for anticancer photodynamic therapy [108]. NO is involved in the expression of biosynthetic genes, monooxygenase (Mono), polyketide synthase (PKS), and O-methyltransferase (Omef), which are involved in hypocrellin A production, and upregulates the expression of transporter genes, major facilitator superfamily (MFS) members, and the ATP-binding cassette (ABC) for hypocrellin A exudation [108]. In addition, the addition of an NO donor (sodium nitroprusside) increases hypocrellin A content in the mycelium by increasing intracellular NO levels [109]. Similar results were found in *A. nidulans*, where the addition of exogenous NO increases mycotoxin production [106]. Endogenous NO also mediates the biosynthesis of antioxidant polyphenols, including inoscavins, phelligridins, davallialactone, and methyldavallialactone [52]. These active substances can be used to treat human diseases caused by oxidative stress, such as cancer, hypertension, neurodegenerative diseases, and autoimmune diseases [52,117]. In *N. crassa*, intracellular NO is actively involved in cellulase production, and cAMP participates in this regulatory effect [107]. An *N. crassa* transcriptome analysis demonstrates that endogenous NO regulates carbohydrate and amino acid metabolism, including pentose and glucuronate interconversion as well as fructose, mannose, galactose, amino and nucleotide sugar, arginine, proline, and tyrosine metabolism [96]. *Preussia* sp. BSL-10, an endophytic fungal strain that produces endogenous NO, indole-3-acetic acid (IAA), and gibberellins (GA4, GA7, GA15, and GA53), promotes edge crop growth and yield [60]. NO biosynthesis has been validated through RT-PCR based on the expression of ent-desaturase oxidase (P450-4), GA14 synthase (P450-1), nitrite reductase (NIRK/NIRS), cytochrome P450 (P450nor), nitrate reductase (NR), NOS-like (NOL) activity, and nitric oxide reductase (QNOR/CNOR) [60]. However, it is unclear whether the production of plant hormones is related to the production of NO [60]. In a co-culture of *I. obliquus* and *P. morii*, the biosynthesis of phenylpropanoids that have antioxidant, anti-inflammatory, antidiabetic, antitumor, and antiviral properties is enhanced, and endogenous NO participates in fungal interspecies interactions [42]. The co-culture of the two fungi triggered the expression of a gene encoding inducible NOS-like protein (iNOSL) in the genome of *I. obliquus*. iNOSL is more responsible for NO production in *I. obliquus* and may serve as important regulators controlling phenylpropanoid production during fungal interspecies interactions [42]. NO biosynthesis is enhanced in two co-cultured fungi, with the subsequent expression of phenylalanine ammonia lyase (PAL) and 4-coumaric-acid–CoA ligase (4CL) and upregulation of styrylpyrone polyphenol biosynthesis in *I. obliquus* [42].

### 3.4. Virulence and Pathogenicity

Pathogenicity refers to the ability of a microorganism to cause disease in a host organism. Virulence is a measure of the severity or harmfulness of a pathogen in damaging a host. In pathogenic microorganisms, NO seems to play a role in both pathogenicity and virulence. In bacteria, endogenous NO is known to regulate toxin biosynthesis and host infection [22]. Some fungi that are pathogenic can cause direct damage to tissues by extending their hyphae into host cells or secreting toxins, and NO plays a role in regulating virulence and interactions with the host organisms [77]. In the hemibiotrophic fungal pathogen *M. oryzae*, endogenous NO regulates spore germination and appressorium formation during the initial stages of infection, and NO removal by using cPTIO (a NO scavenger; 2-(4-carboxyphenyl)-4,4,5,5-tetramethylimidazolin-1-oxy-3-oxide) significantly reduces the formation of barley (*Hordeum vulgare*) lesions [77]. In addition, one study demonstrated that genes encoding enzymes involved in the arginine biosynthetic pathway are essential for pathogenicity in *M. oryzae* [118]. However, the researchers stated that this NO is not generated through an arginine-dependent pathway [118]. In the interaction between the plant host and fungal pathogen, NO appears to be an important mediator for both plant defense and pathogen escape. Because plants produce NO in response to pathogen attacks, pathogens should protect themselves against damage induced by plant-generated NO. Metabolizing NO may be a way for pathogens to escape NO-generated damage. In *M. oryzae*, S-(hydroxymethyl)-glutathione dehydrogenase is involved in metabolizing NO by catalyzing the reduction of S-nitrosoglutathione (GSNO) in the plant [112]. A fungal mutant in which this enzyme is deleted shows increase in sensitivity to exogenous NO in a formaldehyde-containing medium and decrease in both the turgor pressure of spores and appressoria and the toxicity to rice plants, indicating that S-(hydroxymethyl)-glutathione-dehydrogenase-mediated NO metabolism is critical for the virulence of *M. oryzae* [112]. The soil fungus *F. graminearum* recognizes the host before making contact with host plant roots probably by generating intracellular NO [57]. In a phytopathogenic fungus, *B. graminis* f.sp. *hordei*, intracellular NO is a determinant of powdery mildew disease in barley, as it controls fungal appressorium structure formation, thereby affecting host infection [51]. Fungal-pathogen-produced NO can penetrate plant cells, causing host cell death owing to allergic reactions, and this may facilitate the fungal colonization in plant tissue. In the necrotrophic pathogen *B. cinerea*, NO is produced inside the germinating spores and mycelium and in the surrounding medium in vitro [110]. Intracellular NO can diffuse outside the fungal cells, stimulating the fungal colonization of plant tissues [110]. The fungal pathogen *B. ellipsoidum* induces programmed cell death in lilies, and intracellular NO accumulation is observed in both fungal pathogens and plant cells during infection [111]. Fungal-pathogen-produced NO can cause nitrooxidative damage to fungal cellular components. However, a fungus can reduce this stress damage, and this results in maintaining redox balance in infected plant cells, leading to avoiding plant defense stimulation [119].

Endogenous NO production can also influence *A. nidulans* virulence via the regulation of mycotoxin biosynthesis [106]. Mycotoxins seriously threaten human health, and ingesting food contaminated with mycotoxins can cause acute or chronic toxicity to humans and animals. NO increases the ability of *Aspergillus* to produce mycotoxins, which means that NO increases the virulence of this fungus [106].

Although NO has been found to play a crucial role in various aspects of fungal biology, including growth, development, stress response, virulence, pathogenicity, and metabolism, the detailed regulatory mechanisms and downstream targets of NO in fungi are still poorly characterized. cAMP appears to be a putative downstream target of NO in fungi [40,41,46,107]. Endogenous NO can actively promote conidia formation and the production of secondary metabolites in various fungi through the second messenger cAMP [40,41,46]. Studies demonstrate that NO signaling may participate in crosstalk with other signaling pathways, including calcium signaling, ROS signaling, and the mitogen-activated protein kinase (MAPK) cascade [40,120]. Under heat stress, crosstalk between NO and calcium–calmodulin regulates ganoderic acid biosynthesis in *Ganoderma lucidum* [120]. The Ca_2_^+^–NO–cGMP signaling pathway was also found to be involved in zoospore biogenesis in the aquatic fungus *Blastocladiella emersonii* [40]. In *C. minitans*, the MAPK cascade functions upstream of the NO signaling pathway in the conidiation process [121]. In addition, complex crosstalk between NO and ROS signaling pathways also exists in fungi. Both ROS and NO are generated during pre-infection development of a pathogenic fungus, *P. striiformis* f.sp. *tritici*, and participate in inducing spore germination [99]. A study demonstrates that ROS can induce NO generation [122]. In *Aspergillus flavus*, ROS is involved in a fungicide-induced fungal spore death through triggering NO generation, and the addition of exogenous NO can induce spore death in fungal cells in which ROS production is blocked [122]. In a mushroom fungus, *Pleurotus ostreatus*, intracellular NO generated under heat stress causes the reduction in ROS accumulation in the cell by inducing the expression of an oxygenase that slows down cellular respiration, and this eventually leads to enhancing fungal tolerance to heat stress [123].

## 4. Conclusions and Future Perspectives

**Figure 1:**
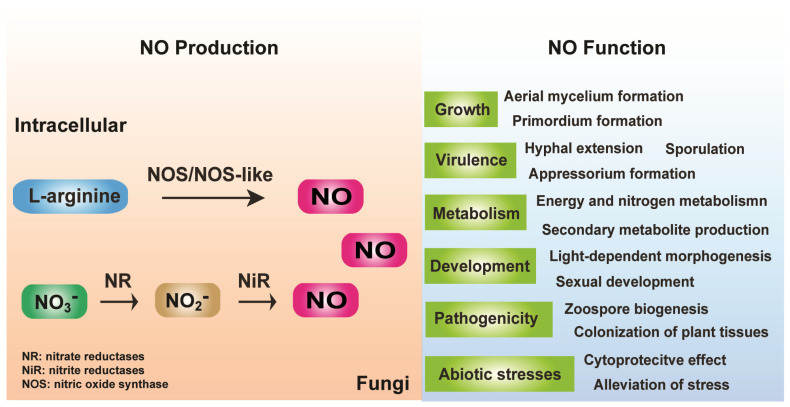
Summary of NO production and function in fungi.

Limited data are available on NO production and its function in fungal cells. Regardless, an increasing number of studies have demonstrated that NO is synthesized in fungal cells and acts as a highly reactive signaling molecule that plays crucial roles in fungal growth and development, metabolic control, virulence enhancement, and environmental adaptation (Figure 1). NO is a universal intracellular regulator of biological functions in all kingdoms of life. However, its biosynthetic pathways do not appear to be well conserved among kingdoms. Compared to the functional analysis of endogenous fungal NO, there is more controversy regarding the biosynthetic mechanisms for fungal NO because NOS with high sequence homology compared to those of mammals, plants, and bacteria has rarely been found in fungal genomes, and nitrite reduction is another mechanism for NO synthesis. NOS-independent synthesis has also been observed in both plants and bacteria. There may be some general mechanisms for NO synthesis that are well conserved among species, but differences in the lifestyle of the species and environmental conditions can result in the generation of various mechanisms. NO can be generated as a byproduct of cellular metabolic pathways, such as mitochondrial respiration and denitrification processes as well as other non-enzymatic reactions. The NO of some plant species is produced via these pathways. This can also be a future research subject for elucidating fungal NO biosynthesis pathways. The production and function of endogenous NO remain poorly understood in fungi, and future studies are required to establish the details of NO biology conservation in all kingdoms of life. 

## Figures and Tables

**Table 1:** Mechanisms for NO synthesis in fungi.

Fungus	Mechanism for NO Synthesis	Experiments for Testing Mechanisms	Reference
*Aspergillus nidulans*	NOS dependent	NOS-like enzyme activity was measured.	[45]
	NO_2_^−^ dependent	NR enzyme activity was measured.	[55]
*Blastocladiella emersonii*	NOS dependent	NOS-like enzyme activity was measured.	[40]
*Blumeria graminis*	NOS dependent	Enzyme activity was inhibited by NOS inhibitors.	[51]
*Colletotrichum coccodes*	NOS dependent	Enzyme activity was inhibited by NOS inhibitors.	[49]
*Coniothyrium minitans*	NOS dependent	NOS-like enzyme activity was measured.	[41]
		Enzyme activity was inhibited by NOS inhibitors.	[50]
*Cylindrocarpon tonkinense*	NO_2_^−^ dependent	Nitrite reductase was expressed and purified. Enzyme activity (NO_2_^−^ reduction to NO) was measured.	[56]
*Flammulina velutipes*	NOS dependent	NOS protein was purified using column chromatography, and activity of purified NOS enzyme was measured.	[37]
*Fusarium graminearum*	NO_2_^−^ dependent	Identification of protein that may possibly induce NR enzyme expression.	[57]
*Fusarium oxysporum*	NO_2_^−^ dependent	Nitrite reductase was expressed and purified. Enzyme activity (NO_2_^−^ reduction to NO) was measured.	[58]
*Ganoderma lucidum*	NO_2_^−^ dependent	NR gene was silenced, and activity of NR was inhibited.	[59]
*Inonotus obliquus*	NOS dependent	Enzyme activity was inhibited by NOS inhibitors.	[52]
*Inonotus obliquus* co-cultured with *Phellinus morii*	NOS dependent	Enzyme activity was inhibited by NOS inhibitors in *I. obliquus*. Genes homologous to constitutive and inducible mammalian NOS were identified, and inducible NOS was expressed in *I. obliquus* during co-culture. Cloned inducible NOS showed enzyme activity.	[42]
*Macrophomina phaseolina*	NOS dependent	Enzyme activity was inhibited by NOS inhibitors, and gene homologous to mammalian NOS was identified.	[54]
*Neurospora crassa*	NOS dependent	Enzyme activity was inhibited by NOS inhibitors.	[48]
	NOS dependent	NOS-like enzyme activity was measured.	[44]
*Phycomyces blakesleeanus*	NOS dependent	NOS-like enzyme activity was measured and inhibited by NOS inhibitors.	[38]
*Pleurotus eryngii* var. *tuoliensis*	NOS dependent	Enzyme activity was inhibited by NOS inhibitors.	[53]
*Preussia* sp. BSL-10	NOS dependent NO_2_^−^ dependent	Genes encoding NOS-like protein, nitrate reductase, and nitrite reductase were expressed.	[60]
*Saccharomyces cerevisiae*	NOS dependent	NOS-like enzyme activity was measured.	[39]
	NOS dependent	Constitutive NOS-like protein was detected by Western blot. Activity of NOS was measured and inhibited by NOS inhibitors.	[36]
	NO_2_^−^ dependent	Nitrite reduction to NO by mitochondrial cytochrome c oxidase under hypoxia condition.	[61]
*Shiraia* sp. Slf14	NOS dependent NO_2_^−^ dependent	Genes homologous to constitutive and inducible mammalian NOS were identified. Cloned inducible NOS showed higher enzyme activity and gene expression under heat stress. Expression of inducible NOS and NR was elevated under heat stress.	[62]
		Transcription level and activity of NOS and NR were elevated.	[46]
*Trichophyton rubrum*	NOS dependent	NOS-like enzyme activity was measured.	[43]

**Table 2:** Endogenous NO function in fungi.

Category	Fungus	Function	Reference
Growth and development	*Aspergillus nidulans*	Reduce conidiation and induce the formation of cleistothecia	[55]
		Light regulation of conidiation	[88]
	*Blastocladiella emersonii*	Controlling zoospore biogenesis	[40]
	*Candida albicans*	Growth promotion and pathogenesis by extracellular vesicles	[97]
	*Colletotrichum coccodes*	Regulation of spore germination	[49]
	*Coniothyrium minitans*	Nitric-oxide-mediated conidiation	[41,50]
	*Neurospora crassa*	Light-induced conidiation and carotenogenesis	[44,48]
		Regulate mycelial development and conidia formation	[95]
		Impacting the growth and development of hyphae (vegetative growth)	[96]
	*Phycomyces blakesleeanus*	Light-induced development of sporangiophores	[38]
	*Physarum polycephalum*	Sporulation	[98]
	*Pleurotus ostreatus*	Primordia formation	[94]
	*Puccinia striiformis* f.sp. *tritici*	Induce spore germination	[99]
Response to stresses	*Aspergillus fumigatus*	Effects of antifungal agent (farnesol) on germination	[100]
	*Ganoderma lucidum*	Heat-stress-induced ganoderic acid levels	[101]
	*Lentinula edodes* and *Grifola frondosa*	Tolerance to superoptimal pH and in nitrogen limitation	[102]
	*Pleurotus eryngii* var. *tuoliensis*	Heat-stress-induced oxidative damage	[53]
		Heat-stress-induced trehalose accumulation	[103]
	*Rhizophagus irregularis*	Enhanced host plant tolerance to low temperature stress by regulating proline accumulation in plant	[47]
	*Saccharomyces cerevisiae*	Cytoprotective effect from heat shock or high hydrostatic pressure	[104]
		Hypoxia signaling	[61,105]
		H_2_O_2_-induced apoptosis	[39]
	*Shiraia* sp. Slf14(w)	Heat-stress-enhanced perylenequinone biosynthesis	[62]
	*Trichophyton rubrum*	Reduction in fungal viability by 420 nm intense pulsed light	[43]
Metabolism	*Aspergillus nidulans*	Mycotoxin production	[106]
	*Ganoderma lucidum*	Methyl-jasmonate-induced ganoderic acid biosynthesis	[59]
	*Inonotus obliquus*	Biosynthesis of antioxidant polyphenols, accumulation of antioxidant phenolic constituents	[52]
	*Inonotus obliquus* and *Phellinus morii*	Increase in level of styrylpyrone polyphenols in fungal interspecific interaction	[42]
	*Neurospora crassa*	Cellulolytic enzyme production	[107]
		Carbohydrate and amino acid metabolism	[96]
	*Preussia* sp. BSL-10	Improve rice plant growth and related gene expression	[60]
	*Shiraia* sp. S9	Hypocrellin A production	[108,109]
	*Shiraia* sp. Slf14(w)	Production of secondary metabolite perylenequinone	[46,62]
Virulence and pathogenicity	*Aspergillus nidulans*	Mycotoxin production	[106]
	*Blumeria graminis*	Influences formation of the appressorium infection structure	[51]
	*Botrytis cinerea*	Saprophytic growth and plant infection	[110]
	*Botrytis elliptica*	Induction of programmed cell death in lily	[111]
	*Fusarium graminearum*	Host recognition and virulence	[57]
	*Magnaporthe oryzae*	Drives plant infection (delays germling development and reduces disease lesion numbers)	[77]
		Conidial germination and appressorium formation (infectious morphogenesis)	[112]

## Data Availability

Not applicable.
